# Supplementary material for: Analysis of the Mitochondrial COI Gene and Genetic Diversity of Endangered Goose Breeds
Source: Genes (Basel). 2024 Aug 6;15(8):1037. doi: 10.3390/genes15081037 (PMC11353847; doi:10.3390/genes15081037)
Supplement: Supplementary file 1 [file genes-15-01037-s001.zip › Table S1.pdf]

Table S1. The basic distribution of 6 rare and endangered goose breeds.

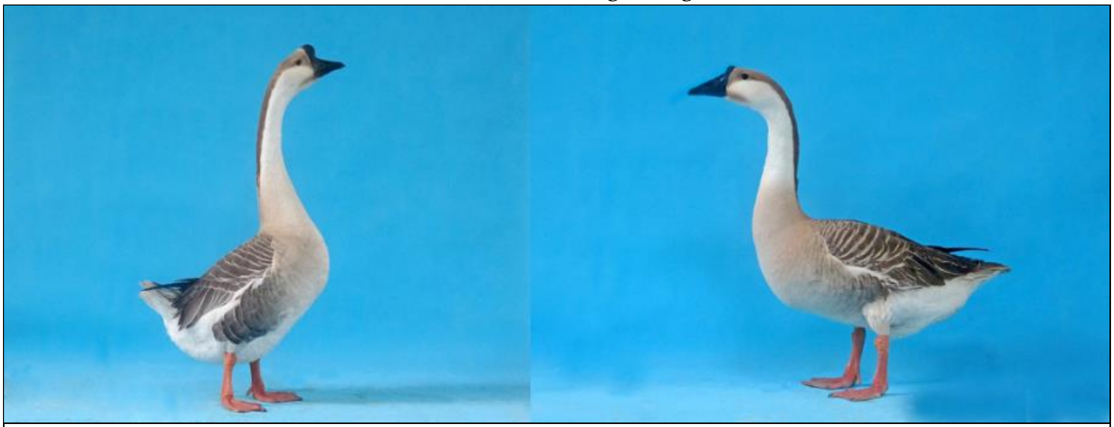

Name: Yan goose（YE）
Genus: *Anser cygnoides*
Origin: Liuan, Anhui
lng and lat: E115°22′,N31°48′
Climatic conditions: The original production area belongs to the transition zone from north subtropical to warm temperate zone, with annual sunshine of 1876~2003.5 hours, total annual precipitation of 1008.5~1545.7 mm in all counties and districts, and evaporation of 1300~1500 mm. The average annual temperature is 16.7~17.9 ℃, and the annual frost-free period averages 211~228 days.<br>The current production area belongs to subtropical humid monsoon climate type, with obvious monsoon and four distinct seasons. Annual sunshine 2107.5 hours, the average annual temperature of 15.6 ℃, the hottest month average 28.1 ℃, the coldest month average 2.7 ℃. The frost-free period is about 240 days, with the first frost starting on November 14th and ending on March 17th of the following year. The dryness is between 0.68~0.90. The annual precipitation is between 1200~1500 mm, with an average of about 1350 mm, and the rainy season lasts about 25 days, with an average precipitation of 200~350 mm, which generally accounts for about a quarter of the annual rainfall. The average annual wind speed is 3~4, and the maximum wind speed is 8~9.
Habits and properties of life: Food quantity is large, fast feeding, strong digestive ability, love to eat the young leaves, stems and buds of plants and grass seeds, also forage other aquatic weeds.

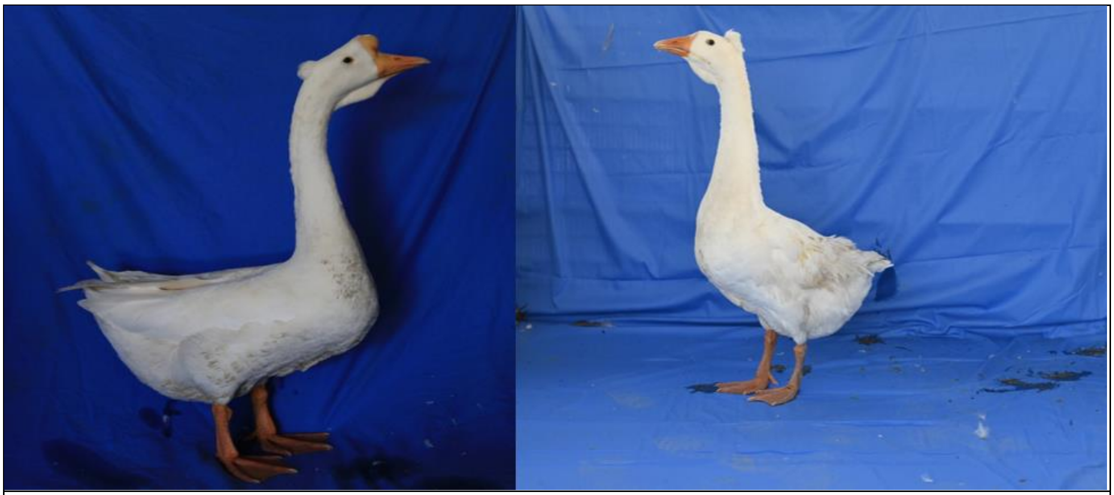

Name: Baizi goose（BZ）
Genus: *Anser cygnoides*
Origin: Jinxiang, Shandong
lng and lat: E116°18′,N35°04′
Climatic conditions:North and south of the two districts of ditches and canals, water supply is sufficient, water and grass are fertile, there are patches of lakes and rivers, green grass grows luxuriantly, the main agricultural production of rice, concentrate, rough fodder is very rich.
Habits and properties of life: Baizi goose are water-loving, gregarious, alert, hardy, rhythmic, and omnivorous.

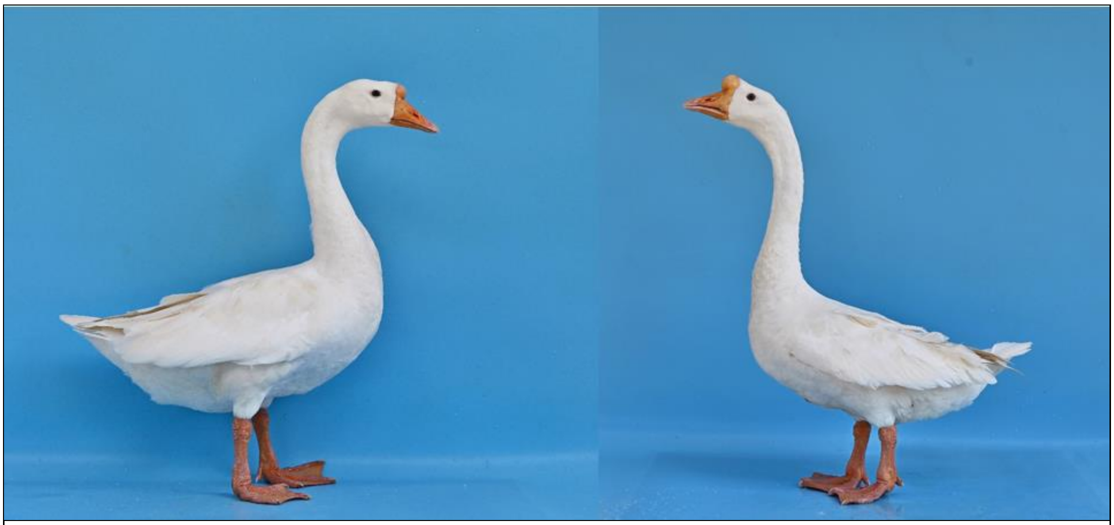

Name: Lingxian goose（LX）
Genus: *Anser cygnoides*
Origin: Yanling, Hunan
lng and lat: E112°57′,N26°03′
Climatic conditions: Central subtropical monsoon humid climate zone, low temperature and cold period is short, early spring warm fast. Different altitudes and different regions, the temperature difference is obvious, both three-dimensional climate characteristics, and microclimate differences. The average annual temperature ranges from 12.1℃ to 17.2℃, the annual sunshine is 1500 hours, the average solar radiation is 86.6 to 105.1 kcal/cm, the average rainfall is 1,761.5 mm, and the frost-free period is 292 days. There are four distinct seasons in a year, with a big temperature difference between day and night, no severe cold in winter and no scorching heat in summer.
Habits and properties of life: The head is medium sized with a smaller sarcoma, which is flattened and inconspicuous in female geese. Neck length medium body feathers white. Bill, sarcoma and tibia, webbing orange, skin yellow, iridescence blue-gray, no pharyngeal pouch in male or female geese. The skin is complete, the muscles are dense and not soft, and the tissues are dense and tough.

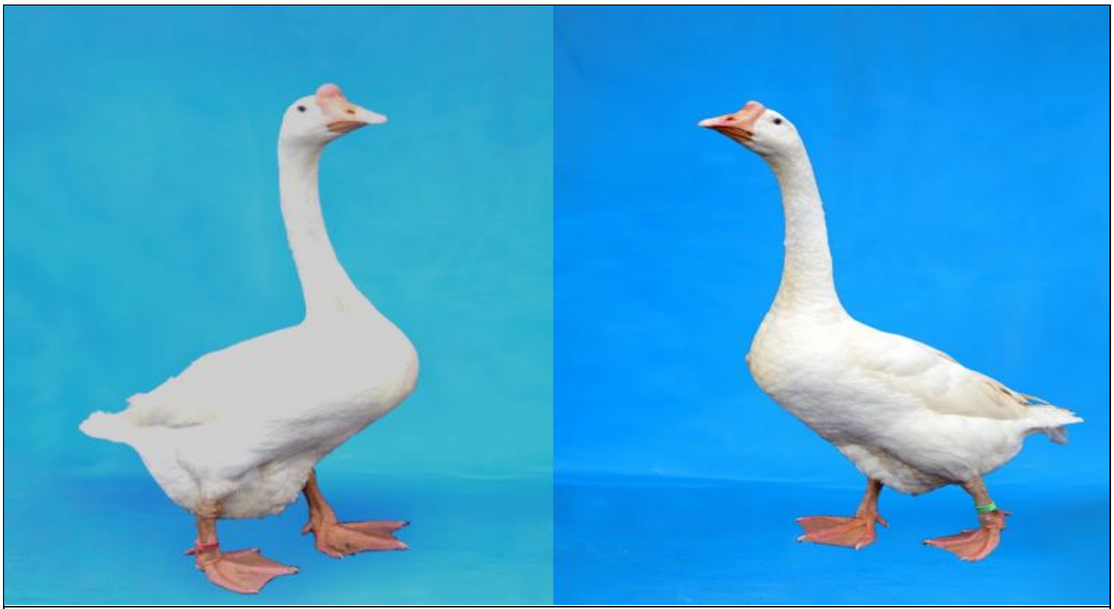

Name: Xupu goose（XP）
Genus: *Anser cygnoides*
Origin: Xupu, Hunan
lng and lat: E110°15′,N28°17′
Climatic conditions: At the northern foot of Xuefeng and the middle reaches of Yuan River, it lies between 110°15′-111°01′ east longitude and 27°19′-28°17′ north latitude, with a length of 110 kilometers from north to south and a width of 72 kilometers from east to west, covering a total area of 3,438 square kilometers. The county is surrounded by high and low in the middle, surrounded by seven forests lingering in the middle of the four major water systems meandering. There is no great cold in winter and no scorching heat in summer, belonging to the subtropical monsoon humid climate zone, with abundant heat, abundant rainfall and four distinct seasons, with an annual average of 1,552 hours of sunshine, an annual average of 1,539 millimeters of precipitation, and an annual average temperature of 16.9℃.
Habits and properties of life: It has the advantages of fast growth rate, high egg production rate, strong foraging power, rough feeding resistance, etc. It has strong adaptability to the natural environment. In addition, it has good liver production performance, large size of goose liver, low cholesterol content, high nutritional value, and it is one of the best comprehensive performance goose breeds for meat and liver in China.

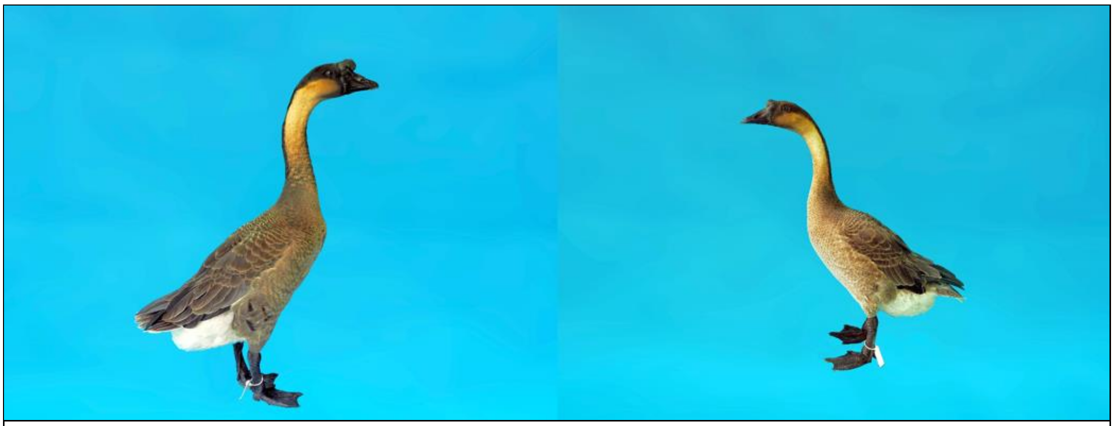

Name: Wuzong goose（WZ）
Genus: *Anser cygnoides*
Origin: Qingyuan, Guangdong
lng and lat: E113°55′,N23°26′
Climatic conditions: Pre-housing, late grazing; pre-housing, mid-term grazing, late-housing fattening; full housing, appropriate combination of grazing, this way although the son of the geese rapid growth, but due to the consumption of concentrates, feeding costs are high, so in the production of geese around the world should be adapted to the local conditions, make full use of the biological characteristics of the geese, to improve economic efficiency. The building requirements of the goose house are very simple (except for the brooder house), as long as it can block the scorching sun, wind and rain can be. But in winter to take windproof measures.
Habits and properties of life: Strong adaptability, foraging ability, especially suitable for southern high temperature, high humidity area feeding. Grass-feeding economic waterfowl, myogastric and cecum are very developed, good at eating grass, greens; rough feeding, easy to fatten, high meat rate. Mother geese have a strong nesting nature.

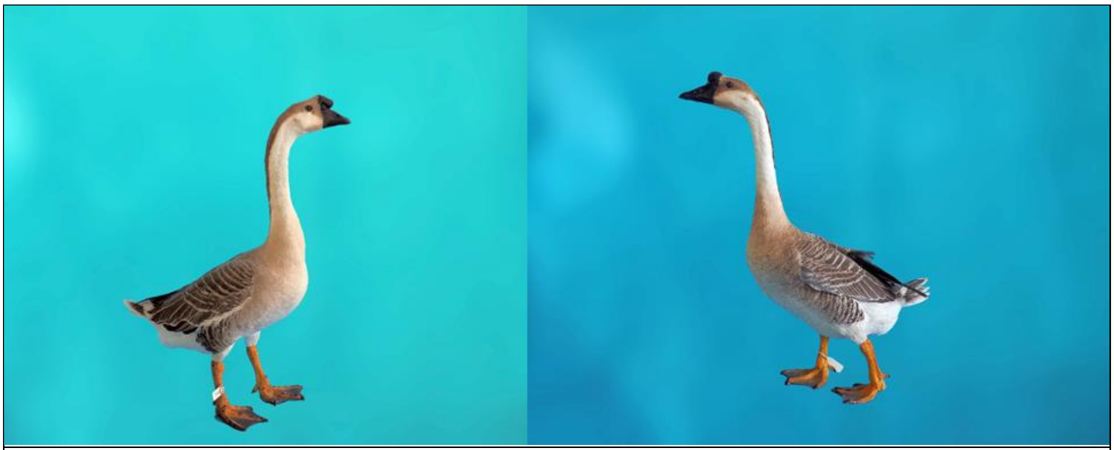

Name: Yangjiang goose（YJ）
Genus: *Anser cygnoides*
Origin: Yangjiang, Guangdong
lng and lat: E111°16′,N21°28′
Climatic conditions: Yangjiang City is located in the southwest coast of Guangdong Province, the city is densely populated with rivers, rainfall catchment area of more than 100 square kilometers of 24 rivers, Yangjiang City, mountains, hills, valleys, islands and other types of landforms, the east, the north, and the south, the western distribution of Ziluoshan, Zhushan, Wangfushan, the central and western East Coast Mountains, Luoqin Mountain, Longgao Mountain; rivers have Mo Yangjiang, Shangyang River, Ruyi Pangyong River, Shou Chang River and Mojiang River tributaries Dabai River and Nalong River, etc.. Rivers include Moyang River, Shangyang River, Ruguang River, Shouchang River and tributaries of Muojiang River, such as Daba River and Nalong River. The terrain slopes from north to south and is surrounded by mountains and the sea, with Tianlu Mountain in the northeast and Yunwu Mountain in the northwest. And with abundant sunshine and rainfall, it has a good geographical environment, soil, water and grassland. Yangjiang belongs to the subtropical monsoon climate zone, with obvious oceanic climate, long light hours and abundant heat; abundant rainfall and long rainy season; mild climate and long frost-free period. This geographical environment and climate is the best place for Yangjiang yellow maned goose rearing and production.
Habits and properties of life: Early maturity, easy fattening, tender meat.
